# Characteristics of Plasmablast Repertoire in Chronically HIV-Infected Individuals for Immunoglobulin H and L Chain Profiled by Single-Cell Analysis

**DOI:** 10.3389/fimmu.2019.03163

**Published:** 2020-02-11

**Authors:** Hongyan Liao, Song Li, Yangsheng Yu, Yinshi Yue, Kaihong Su, Qin Zheng, Nenggang Jiang, Zhixin Zhang

**Affiliations:** ^1^Department of Laboratory Medicine, West China Hospital, Sichuan University, Chengdu, China; ^2^Department of Pathology and Microbiology, University of Nebraska Medical Center, Omaha, NE, United States; ^3^Department of Chemotherapy, Cancer Center, Qilu Hospital of Shandong University, Jinan, China; ^4^Internal Medicine, University of Nebraska Medical Center, Omaha, NE, United States; ^5^Eppley Research Institute, University of Nebraska Medical Center, Omaha, NE, United States; ^6^State Key Laboratory of Biotherapy, Ministry of Education Key Laboratory of Birth Defects, Department of Pediatrics, West China Second University Hospital, Sichuan University, Chengdu, China

**Keywords:** human immunodeficiency virus, plasmablast, repertoire, variable region, single-cell PCR

## Abstract

Characterization of the diversified immunoglobulin (Ig) repertoire may provide insight into pathways that shape an efficient antibody (Ab) repertoire for immune response against human immunodeficiency virus (HIV) infection. This study aimed to profile characteristics of the plasmablast repertoire during chronic HIV infection. Ig variable regions of plasmablasts from both chronically HIV-infected donors (HIVDs) previously treated with antiretroviral therapy (ART) and healthy donors (HDs) were amplified by single-cell PCR to establish the basis for further repertoire analysis. We compared the plasmablast repertoires expressed in multiple chronically HIVDs after ART treatment cessation and HDs. We also examined the non-productive repertoire to identify the indication of the immediate products of the rearrangement machinery without an impact of selection during HIV infection. We found multiple differences between the productive repertoires of HIVD and HD subjects, including biased usages of VH3-49, VH1-2, VH3-33, VH3-74, and VH5-51 in VH and D1-7, D1-14, D1-20, and D5-5/18 in D segments in the HIVD group, as well as shorter and preferential glycine usages in CDRH3 regions. Gene selections were also detected in light chains. Notably, differences between productive rearrangements of HIVDs and HDs outnumbered those between productive and non-productive rearrangements within HIVDs. HIV infection may exert a dominant impact on the development of the plasmablast repertoire. The impact of selection is of limited significance in shaping the plasmablast repertoire. Overall, the data indicate that the environment in which the plasmablasts live can affect the distribution of the VH and VL genes in the repertoire and the amino acid compositions of the expressed Abs.

## Introduction

Elicitation of broadly neutralizing antibody (bNAb) of interest via vaccination is recognized as the ultimate approach for combatting human immunodeficiency virus (HIV) infection (1). An enormously diversified and non-self-reactive antibody (Ab) repertoire is the basis of a potent Ab response. The combinatorial choice of variable (V), diversity (D) (for heavy chain only), and joining (J) segments on immunoglobulin (Ig) heavy (IgH) and light (IgL) chains contributes to the diversity of the functional Ab repertoire. The diversified Ig repertoire is generated by molecular events before antigen (Ag) encounter and, later, through somatic hypermutation and class switch recombination during Ag-dependent germinal center reactions (2). Since the introduction of antiretroviral therapy (ART), the life expectancy of HIV-infected individuals has increased remarkably (3). However, there are significant gaps in our knowledge of Ig repertoires during HIV infection. Thorough profiling involving a repertoire-wide analysis to elucidate the underlying sophisticated mechanisms by which the repertoire in the context of HIV infection is ontogenetically developed and shaped remains an unmet need. A better understanding of Ig repertoire development would provide striking insights for bNAb elicitation.

Plasmablasts are proliferative Ab-producing cells that readily secrete Abs and are largely derived from preexisting memory cells (4). Therefore, plasmablast repertoire characteristics may reflect the effect of the current ongoing infection more precisely than other B cell subsets. The study of this subpopulation of B cells may answer critical questions regarding the origin, complexity, and evolution of Ab responses. Human plasmablasts have been studied extensively to monitor B cell responses and to generate disease-specific monoclonal Abs (mAbs) (5, 6). We have also demonstrated increased plasmablast percentages and potent systemic responses during chronic HIV infection (7). However, no study of human plasmablasts has addressed the genomic complexity of Ig repertoires in chronic HIV infection.

The current study aimed to characterize the plasmablast repertoire in chronic HIV infection. We performed single-cell PCR for amplification and further compared the plasmablast repertoires expressed in multiple chronically HIV-infected donors (HIVDs) and healthy donors (HDs). Productive rearrangements may be positively or negatively selected, as they are capable of interacting with Ags (8). Nonproductive rearrangements either do not encode an Ig receptor, code for an Ig receptor that cannot engage with Ag, or code for an Ig receptor that is not subjected to positive or negative selection. These rearrangements were also included in the comparative analysis to evaluate the impacts of the molecular mechanisms that occur before HIV Ag encounter in shaping the plasmablast repertoire. Collectively, our results from this broad comparison provide insights into how the plasmablast repertoire develops during chronic HIV infection.

## Materials and Methods

### Human Specimens

Peripheral blood samples from seven chronically HIVDs were obtained from the Specialty Care Center at the University of Nebraska Medical Center (UNMC), Omaha, Nebraska. All study subjects were free from other ongoing infections and were chronically infected (>2 years) with a stable CD4^+^ T-cell count after ART. All patients were previously treated with ART but currently off ART with the exception of one subject. Serum neutralization titers for the HIV-infected individuals were determined, and none of the donors displayed neutralization abilities against HIV viral isolates (data not shown). Seven individuals were analyzed as controls, which were all healthy people without ongoing infections and chronic diseases, and were matched with the HIVDs by age, gender, and race. The median age was 34 (ranging from 23 to 54 years old) for HIV group and 40 (ranging from 35 to 51 years old) for the controls. CD4^+^ T-cell count and viral load in all study subjects were analyzed. Demographics of all study subjects are shown in Supplementary Table 1.

### Cell Preparation, Sorting, and Lysate Preparation

Peripheral blood mononuclear cells (PBMCs) were purified by Ficoll gradient centrifugation from 20 ml of peripheral blood samples. PBMCs were stained according to the procedure reported previously (7). After staining, HIV-positive samples were fixed with 4% formaldehyde (Sigma-Aldrich, St. Louis, MO, USA) for 30 min at room temperature (RT) to eliminate live virus. Circulating plasmablasts were identified as CD3^−^CD19^low^CD20^low^CD27^hi^CD38^hi^ cells by FACS Aria (BD Biosciences) (Supplementary Figure 1A) with the frequency in CD19^+^ B cells shown in Supplementary Figure 1B. The cells were sorted into 96-well PCR plates at 1 cell/well with 10 μl of lysis buffer (10 mM Tris-HCl pH8.0, 10 U RNasin (Promega, Madison, WI, USA). Four plates, i.e., 384 single cells, were sorted from each subject. The sorted single cells were directly subjected to RT-PCR amplification.

### Amplification of Ig Genes by Single-Cell PCR

The 96-well PCR plates containing sorted cells were incubated at 65°C for 4 h to reverse the cross-linking. RT-PCR was carried out using the QIAGEN OneStep RT-PCR kit following the manufacturer's instructions. Three rounds of PCR reactions were used to amplify IgH, Igκ, or Igλ genes from single cells as previously described (9). Briefly, Ig genes from plasmablasts were amplified by a 50-cycle one-step RT-PCR, a 50-cycle nested PCR, and a third round PCR for further subcloning and sequencing. PCR efficiencies ranged from 30 to 60%. All PCR products were purified (QIAGEN) and sequenced (Macrogen). Sequencing reactions were performed using BigDye® v3.1 (Life Technologies, Applied Biosystems) per the manufacturer's protocol. Thermal cycling conditions are as follows: 96°C hold for 2 min, followed by 25 cycles of 96°C for 15 s, 50°C for 5 s, and 60°C for 2 min. Sequence detection was performed by capillary electrophoresis on a 3730xl Genetic Analyzer (Life Technologies, Applied Biosystems) using a 50 cm array, the Long DNA sequencing module (LongSeq50_POP7), and the KB analysis protocol (KB basecaller) with the default instrument settings. Post-detection, raw signal data are initially processed on the 3730xl Genetic Analyzer computer using Sequencing Analysis v5.3.1 (Life Technologies, Applied Biosystems). Primer sequences for PCR and sequencing are described in Supplementary Table 2.

### Sequence Analyses

Numbers of unique IgH and IgL variable (V) region sequences obtained from each study subject are displayed (Supplementary Table 3). The overall sequences were analyzed using the ImMunoGeneTics information system (IMGT)/V-QUEST program (http://www.imgt.org/IMGT_vquest/share/textes/) (10) to assign V, DH, and J genes. A productive rearrangement was considered if the VDJ junction maintained the reading frame into the JH segment. A rearrangement was considered non-productive if it was out of frame or introduced a stop codon during the rearrangement at a junction. Each rearrangement was unique as defined by gene usage and V–D and D–J junction analysis according to IMGT/V-QUEST and manual analysis to validate the most appropriate allelic variant vs. a mutation. Polyclonal sequences were determined if groups of sequences were with the same VH gene, the same JH gene, the same junction length, and CDRH3 amino acid sequences of high identity. Identical sequences due to polyclonal expansion were removed (Supplementary Table 4).

### Statistical Analyses

Statistical analyses were performed using the GraphPad Prism 5.0 (GraphPad Software, Inc., San Diego, CA). The chi-square test or two-tailed Fisher's exact test with 95% confidence intervals was used to assess the differences between the repertoires. Two-sided *p*-values of ≤ 0.05 were considered statistically significant.

## Results

### Differential Recombination and Germline Gene Utilization of Variable Regions at H and L Chain Loci

**Figure 1:**
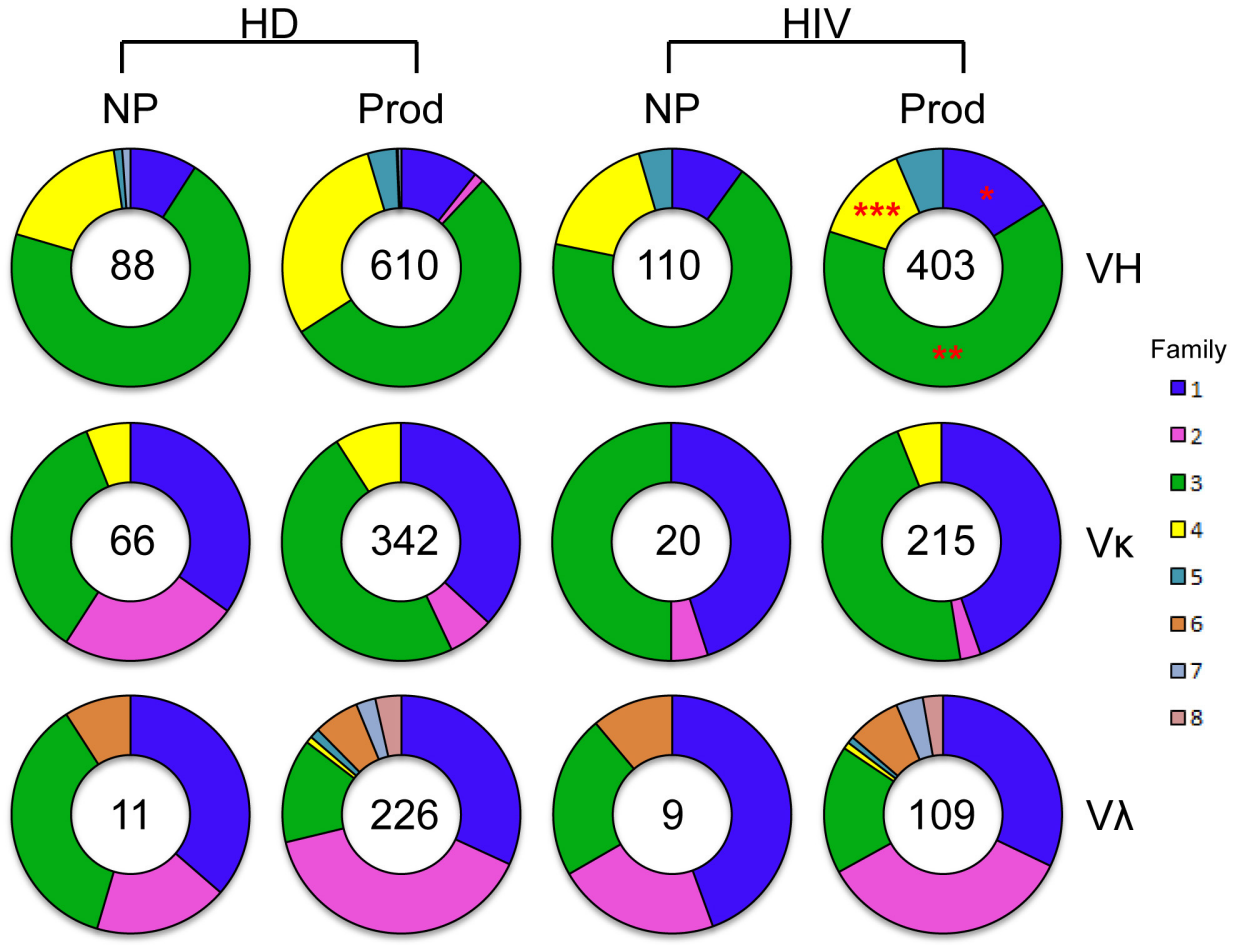
VH-, Vκ-, and Vλ-family distribution of RT-PCR–amplified sorted plasmablasts analyzed with ImMunoGeneTics information system (IMGT)/V-Quest. Families are color-coded. The size of the colored area corresponds to the percent out of the total number of sequences, as is indicated in the center of the pie graphs. Differences in the gene family distributions were evaluated between productive repertoires of healthy donors (HDs) and HIV-infected donors (HIVDs) by chi-square test. A significant difference was considered when two-sided *p*-value < 0.05. *p < 0.05; **p < 0.01; ***p < 0.0001.

To evaluate the efficiency of Ig amplification, we examined the total number of both non-productive and productive V genes obtained from a single plasmablast by PCR (Figure 1). A total of 698 VH sequences were amplified from HDs, consisting of 610 productive (87%) genes, while 513 VH sequences were obtained from HIVDs, with 403 (79%) being productive. VH3 was the largest family used in the productive repertoires of both HDs and HIVDs. The frequencies of VH3 and VH1 gene usage were higher in the productive repertoire from HIVDs (*p* = 0.0022 and *p* = 0.0125, respectively), while that of VH4 was lower (*p* < 0.0001). Thus, VH1 was the second most frequently used gene family in HIVDs, while HDs used VH4 more often. We identified a similar distribution of the Vκ and Vλ gene families in the HD and HIVD groups. Therefore, the major difference in gene family distribution was reflected in VH gene usage. To gain information about the variations among the individuals, the distribution of the single gene families for each individual was also shown (Supplementary Figure 2). U13 and U78 preferentially used VH4 compared to any other HIVDs except for HIV7. U111 used Vκ2 more frequently than all HIVDs. There was no significant difference identified for the usage of other variable gene families.

### Biased H Chain Gene Usages

**Figure 2:**
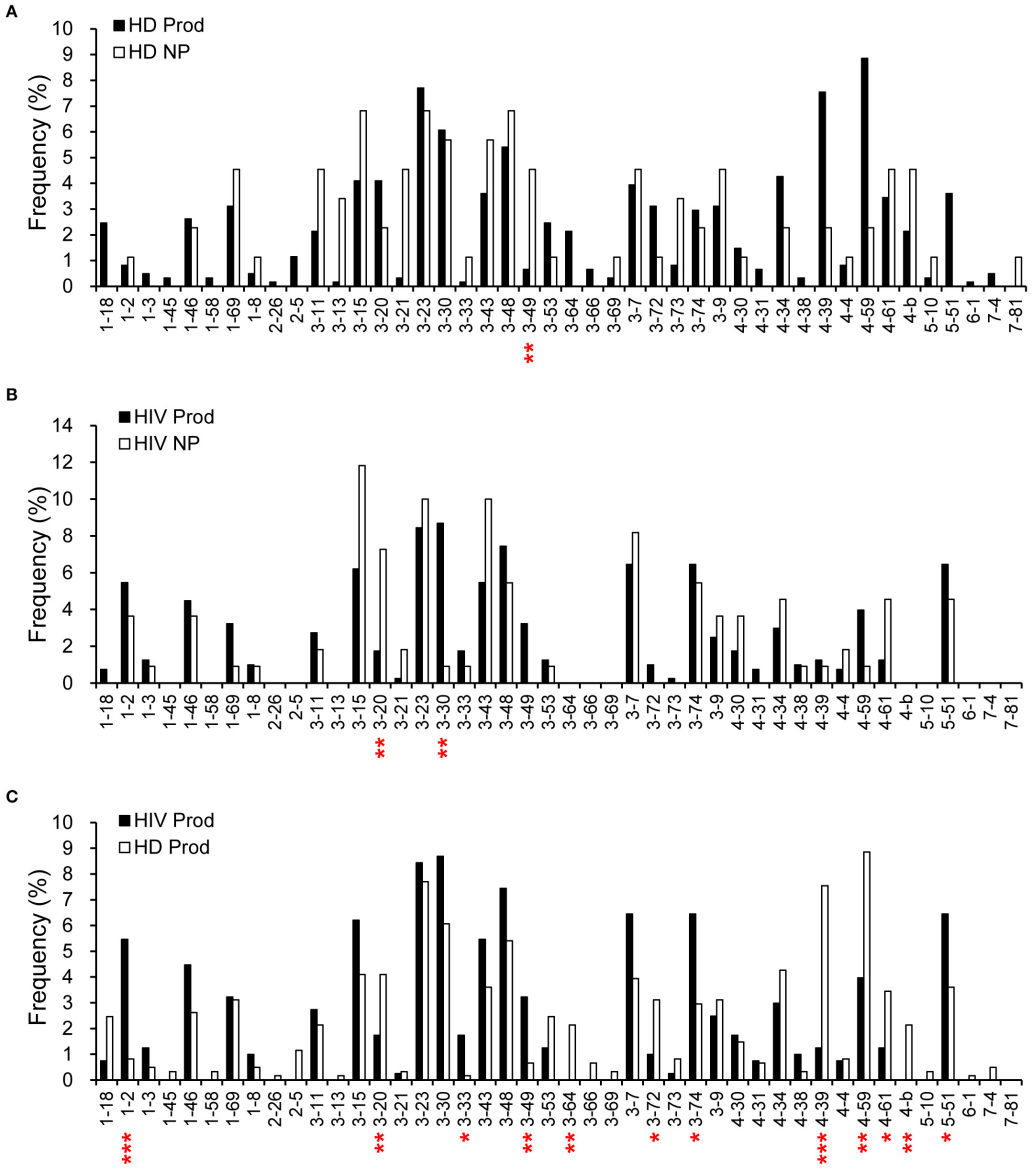
Comparisons of H chain gene utilization between productive (Prod) and non-productive (NP) repertoires of HDs (HD) **(A)** and HIVDs **(B)**, respectively, and in productive repertoires between HDs and HIVDs **(C)**. Chi-square or Fisher's exact tests were performed. A significant difference was considered when two-sided *p*-value < 0.05. *p < 0.05; **p < 0.01; ***p < 0.0001.

We further compared the VH gene frequencies in the plasmablast repertoires. VH3-49 was more frequently used (*p* = 0.0076) in the non-productive repertoire from HDs than in the productive counterpart (Figure 2A). For HIVDs, we found a tendency to use VH3-30 (*p* = 0.0088) and a decreased VH3-20 usage (*p* = 0.0059) (Figure 2B) in shaping the productive repertoire. Furthermore, VH1-2, VH3-33, VH3-49, VH3-74, and VH5-51 were used at higher frequencies in the productive gene rearrangements obtained from HIVDs than in those from HDs (*p* < 0.0001, *p* = 0.0129, *p* = 0.0041, *p* = 0.0110, *p* = 0.0485, respectively; Figure 2C), suggesting a bias favoring their recombination. On the contrary, VH3-20, VH3-64, VH3-72, VH4-39, VH4-59, and VH4-b appeared to be negatively selected in the productive repertoire of HIVDs (*p* = 0.0426, *p* = 0.0077, *p* = 0.0451, *p* < 0.0001, *p* = 0.0023, *p* = 0.0077, respectively) (Figure 2C). Thirteen (VH1-45, VH1-58, VH 2-26, VH2-5, VH3-13, VH3-64, VH3-66, VH3-69, VH4-b, VH5-10, VH6-1, VH7-4, and VH7-81) of the 39 known functional VH genes were not detected in HIVDs (Figure 2C), indicating a remarkably reduced diversity of the plasmablast repertoire during HIV infection. Therefore, plasmablasts from chronically HIVDs may be less efficient than those from HDs at recombining human V(D)J genes, or some of the human VH genes may be functionally inactive or positively selected during chronic HIV infection.

### D Gene Utilization at H Chain Locus

**Figure 3:**
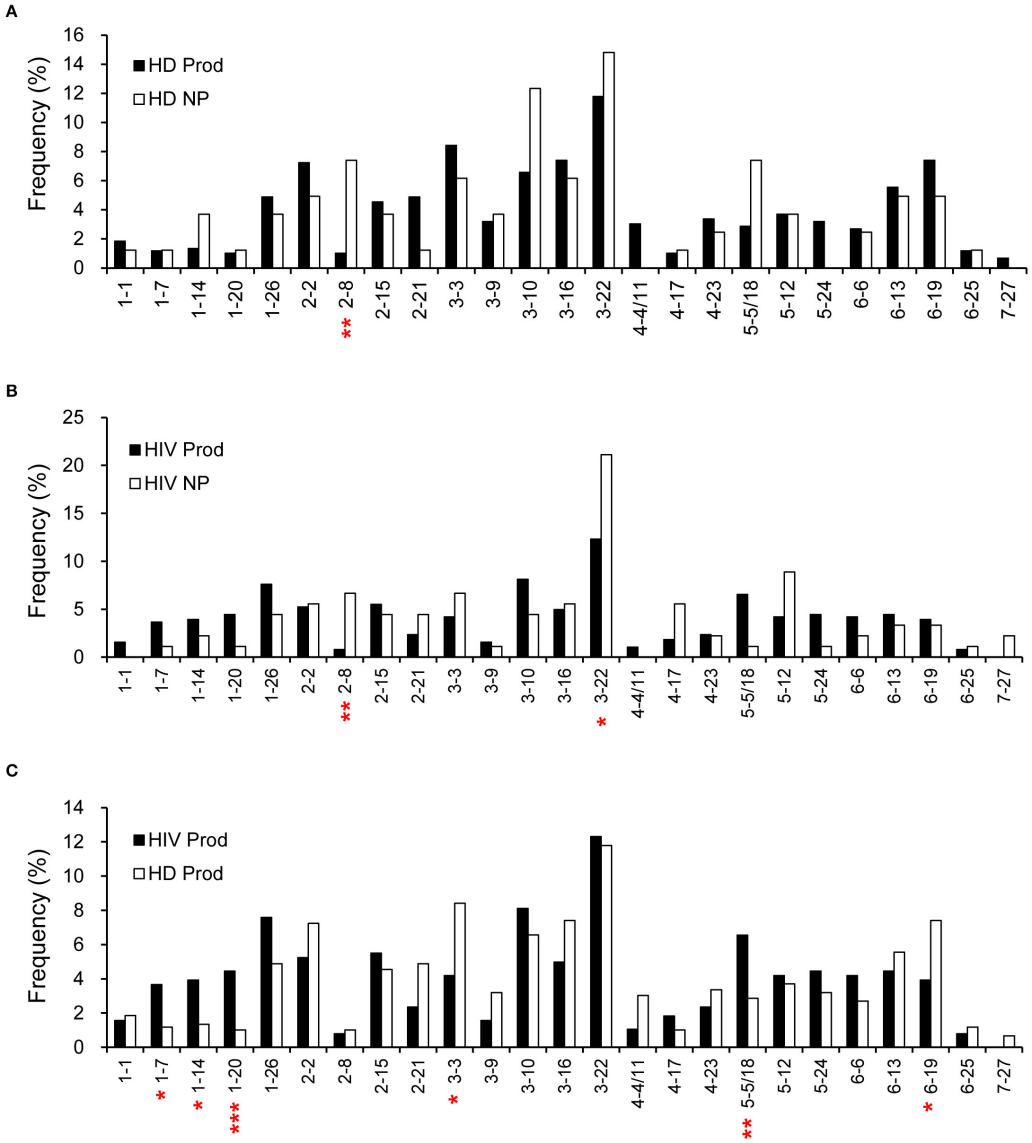
Frequencies of identifiable D segments in productive (Prod) and non-productive (NP) rearrangements of HDs **(A)** and HIVDs **(B)**, and productive repertoires between HDs and HIVDs **(C)**. Chi-square or Fisher's exact tests were performed. A significant difference was considered when two-sided *p*-value < 0.05. *p < 0.05; **p < 0.01; ***p < 0.0001.

According to the IMGT database analysis, there were 594 rearrangements from the productive (97%) and 81 from the non-productive (92%) repertoires of HDs for which a D segment was assigned. Meanwhile, 382 rearrangements from the productive (95%) and 90 from the non-productive (82%) repertoires of HIVDs were successfully matched to a D segment. D2-8 was the only D segment gene infrequently used (*p* = 0.0013) in the productive repertoire of HDs (Figure 3A). Interestingly, while most D genes were used at a comparable frequency, D2-8 and D3-22 were the only two genes used less frequently in the productive plasmablast repertoire of HIVDs (*p* = 0.0012, *p* = 0.0457, respectively) compared to usage in the non-productive repertoire (Figure 3B). In contrast, multiple D genes were differentially used based on the comparison of the productive repertoires of HIVDs and HDs. The former used D1-7, D1-14, D1-20, and D5-5/18 at significantly higher frequencies (*p* = 0.012, *p* = 0.0154, *p* = 0.0008, *p* = 0.0089, respectively), whereas D3-3 and D6-19 were negatively selected (*p* = 0.0125, *p* = 0.0276, respectively) during HIV infection (Figure 3C).

**Figure 4:**
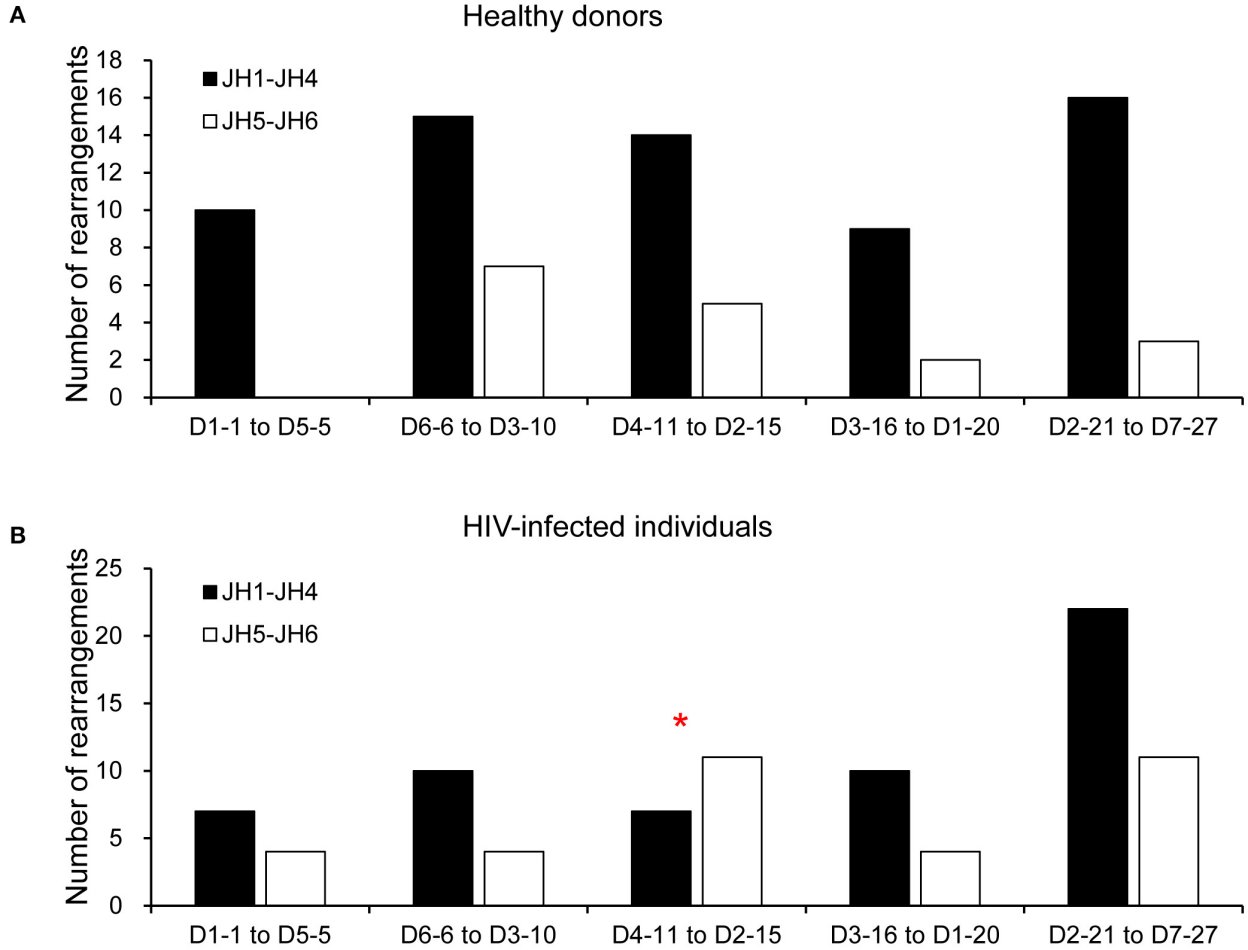
Pairing of IGHD and IGHJ segments in the non-productive repertoire of healthy donors **(A)** and HIVDs **(B)**. Black bars indicate D proximal JH genes (JH1, JH2, JH3, and JH4), and gray bars indicate D distal JH genes (JH5 and JH6). The D genes are divided into five groups according to their position in the locus (D1-1 to D5-5 are the most JH distal, and D2-21 to D7-27 are the most JH proximal). Only the 81 rearrangements from HDs and 90 rearrangements from HIVDs for which a D segment could be assigned were analyzed. * Significant (*p* < 0.05) difference when compared with controls.

To determine whether there was a bias for particular D-JH rearrangements, we analyzed the combinatorial preferences of the non-productive repertoires (Figure 4). Only the 81 non-productive rearrangements from HDs and 90 from HIVDs for which a D segment could be assigned were included in this analysis. Interestingly, 5′ or 3′ D segments (JH distal or JH proximal) were indiscriminately paired with 5′ or 3′ JH segments in the non-productive repertoires of both HDs and HIVDs, except that there was a higher frequency of pairing of the intermediate D segments (D4-11 to D2-15) (*p* = 0.0304) with 3′ JH segments (JH1 to JH4) in HIVDs (Figure 4B).

### J Gene Utilization at H Chain Locus

**Figure 5:**
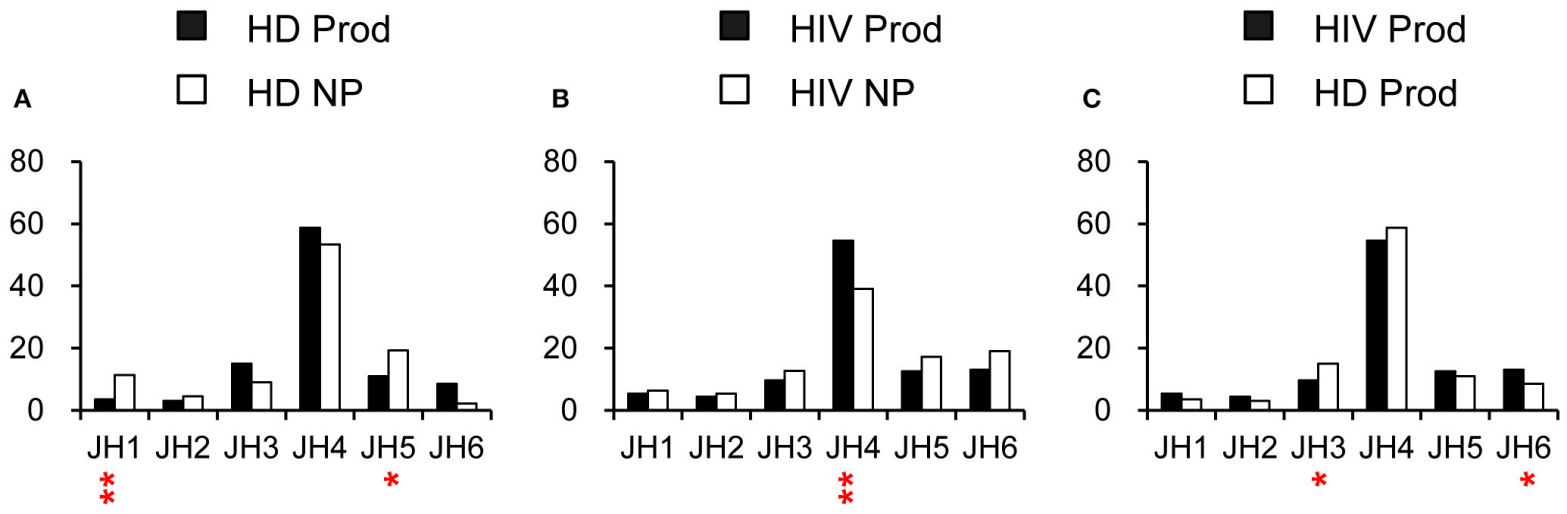
J utilization of H, κ, and λ locus in HDs and HIVDs. HD **(A)** and HIVD **(B)** non-productive (NP) and productive (Prod) rearrangements correspond to the VH repertoires. For all three chains, frequencies of J gene usages were also compared between the productive repertoires of HIVDs and HDs **(C)**. Chi-square or Fisher's exact tests were used, and a significant difference was considered two-sided *p*-value < 0.05. *p < 0.05; **p < 0.01.

To further evaluate the impact of chronic HIV infection on the recombination and selection of complementarity determining region 3 (CDR3), we analyzed the distribution of J genes from the H chain locus. JH4 was the most commonly used in all repertoires (Figures 5A–C). JH6 was found at a significantly higher frequency in the productive repertoire of HIVDs than in that of HDs, while JH3 was negatively selected (*p* = 0.0205, *p* = 0.0128, respectively; Figure 5C) in HIVDs. Therefore, HIV infection seemed to favor the positive selection of more distal JH gene segments.

### κ Repertoire Analysis

**Figure 6:**
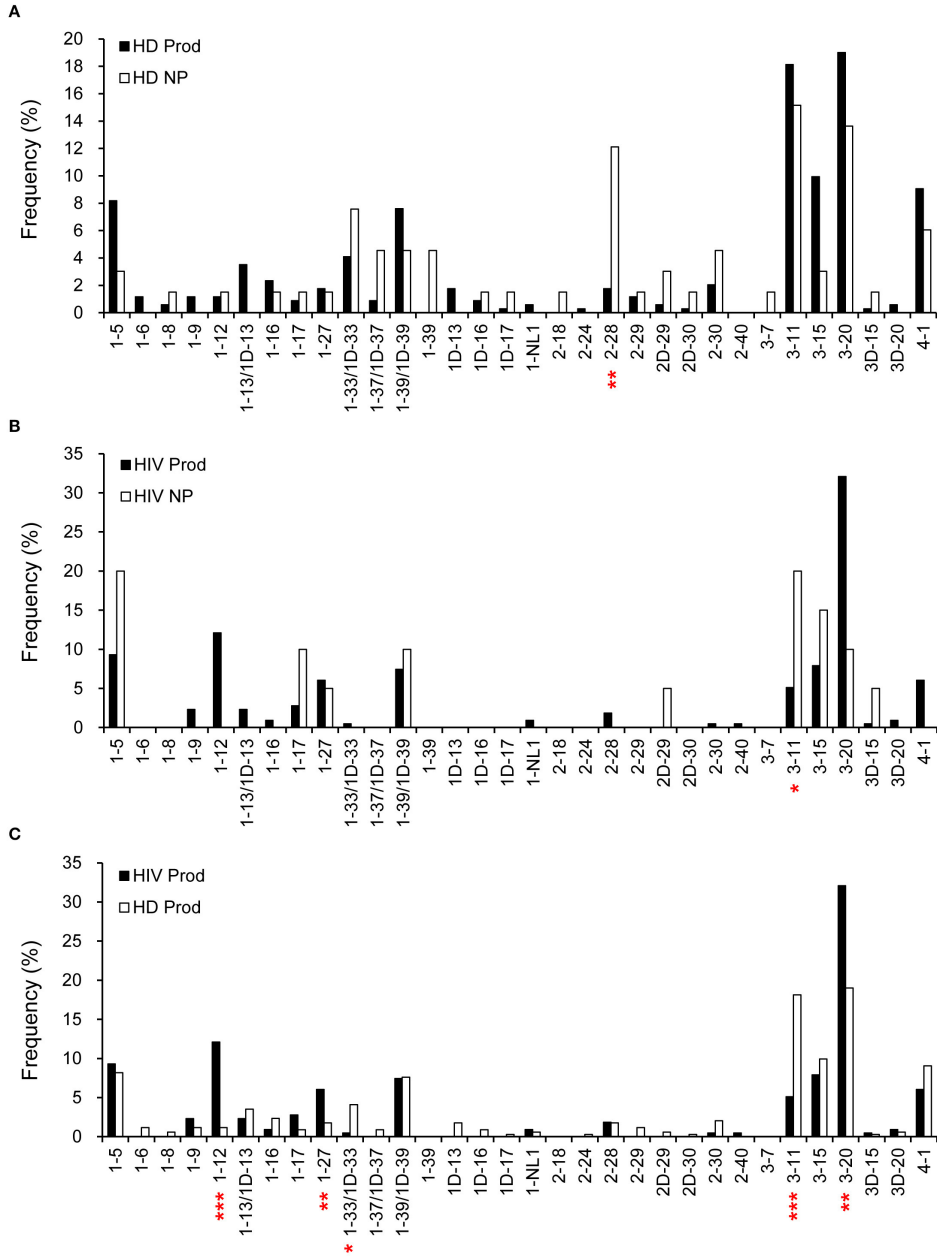
κ gene utilization in HDs and HIVDs. Only Vκ genes expressed in either HDs or HIVDs are displayed. Frequencies of Vκ in productive (Prod) and non-productive (NP) rearrangements of HDs **(A)** and HIVDs **(B)**, and productive repertoires between HDs and HIVDs **(C)**. Chi-square or Fisher's exact tests were performed. A significant difference was considered when two-sided *p*-value < 0.05. *p < 0.05; **p < 0.01; ***p < 0.0001.

Overall, there were 342 productive (83.8%) and 66 non-productive VκJκ sequences obtained from HDs (Figure 1). Vκ2-28 was negatively selected in the productive repertoire of HDs (*p* = 0.0004; Figure 6A). For HIVDs, 235 VκJκ sequences were obtained, of which 91.5% were productive rearrangements (Figure 1). There was negative selection against Vκ3-11 in the productive repertoire of HIVDs (*p* = 0.0335; Figure 6B). Preferential usages of Vκ1-12, Vκ1-27, and Vκ3-20 (*p* < 0.0001, *p* = 0.0083, *p* = 0.0005, respectively) and decreased usages of Vκ1-33/1D-33 and Vκ3-11 (*p* = 0.0211, *p* < 0.0001, respectively) were observed in the productive repertoire of HIVDs compared to that of HDs (Figure 6C).

In the productive repertoire of HIVDs, Jκ5 was negatively selected (*p* = 0.0044; Supplementary Figure 3B). Unlike the H chain, the κ chain was more prone to use the proximal Jκ1 in the productive repertoire of HIVDs (*p* < 0.0001; Supplementary Figure 2C), with notably lower frequencies of Jκ3 and Jκ5 usage (*p* = 0.0129, *p* = 0.0163, respectively; Supplementary Figure 3C). For HDs, the productive repertoire used significantly more Jκ1 and less Jκ3 than the nonproductive (*p* = 0.0395 and *p* = 0.0058 respectively; Supplementary Figure 3A).

### λ Repertoire Analysis

**Figure 7:**
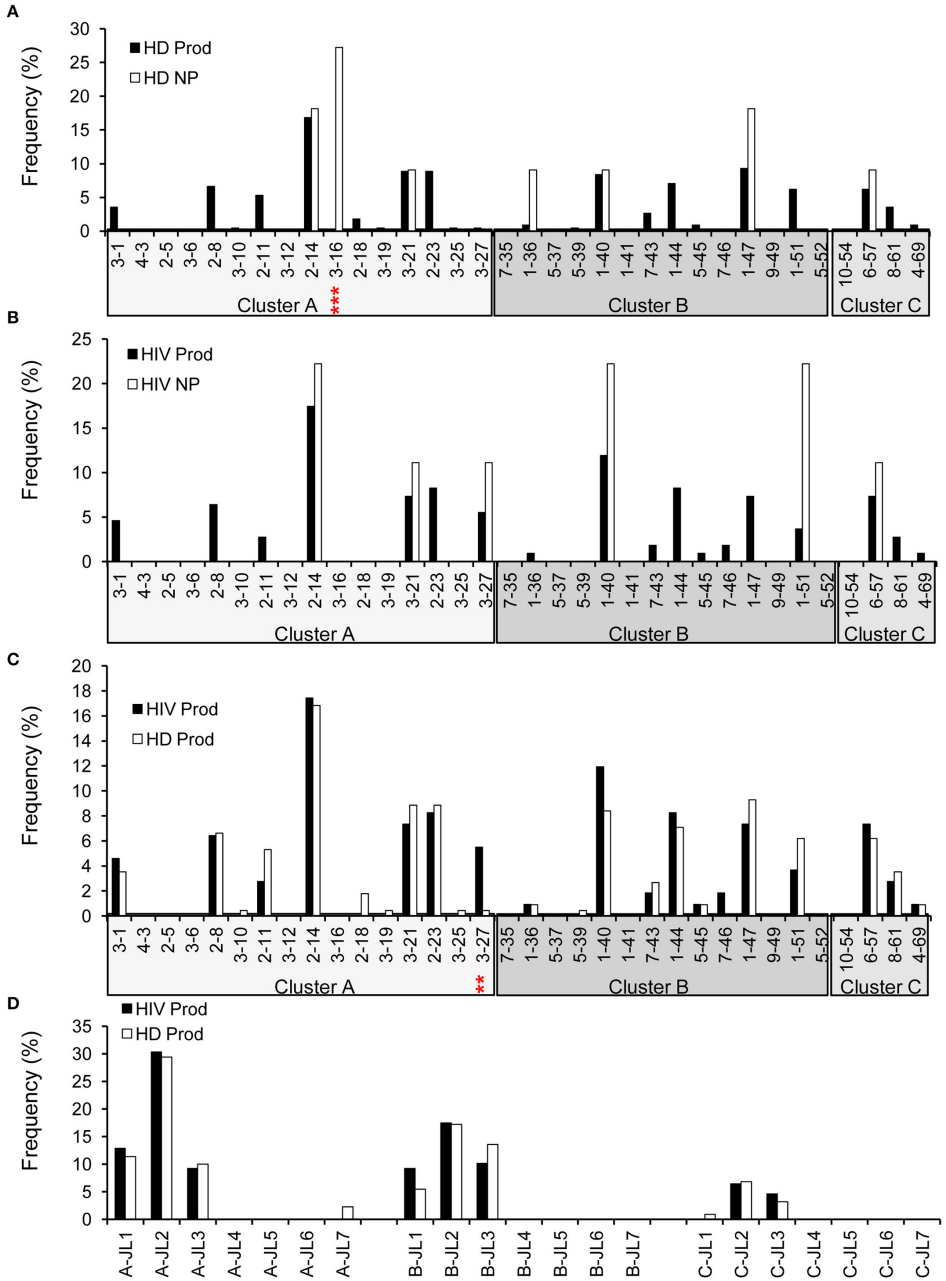
λ gene utilization in HDs and HIVDs. Only Vλ genes expressed in either HDs or HIVDs are displayed. Frequencies of Vλ in productive (Prod) and non-productive (NP) rearrangements of HDs **(A)** and HIVDs **(B)**, and productive repertoires between HDs and HIVDs **(C)**. Genes are displayed in their order in Vλ locus from Jλ proximal cluster A to Jλ distal cluster C. **(D)** Display Jλ utilization by Vλ cluster in productive repertoires of HIVDs and HDs. Chi-square or Fisher's exact tests were performed. A significant difference was considered when two-sided *p*-value < 0.05. **p < 0.01; ***p < 0.0001.

Early studies have established that there are about 34 functional variable genes arranged in three clusters designated J-proximal clusters A and B and J-distal cluster C at the human λ locus (11). We obtained 226 productive (95.4%) and 11 non-productive VλJλ sequences from HDs (Figure 1). For HDs, the distributions of clusters in both the productive and non-productive repertoires were ordered as cluster A > cluster B > cluster C (Figure 7A). The productive repertoire of HDs utilized 11 of 16 (69%) cluster A genes in 117 of 226 sequences (53%), 8 of 11 (73%) cluster B genes in 80 of 226 sequences (35%), and 3 of 4 cluster C genes in 24 of 226 sequences (11%). The productive repertoire of HDs showed a preferred usage of Vλ3-16 among cluster A genes (*p* < 0.0001, Figure 7A). The productive repertoire of HIVDs used 7 of 16 (44%) cluster A genes in 52 sequences (48%), 8 of 11 (73%) cluster B genes in 43 sequences (39%), and 1 of 4 (25%) cluster C genes in 14 sequences (13%). This distribution pattern was more variable than that of the non-productive repertoire, as the latter utilized only 3 of 16 cluster A genes, 2 of 11 cluster B genes, and 1 of 4 cluster C genes. There was no evidence of significantly differentially used genes between the productive and non-productive repertoires of HIVDs (Figure 7B), possibly due to the limited numbers of sequences obtained. Notably, Vλ3-27 in cluster A was positively selected in the productive repertoire of HIVDs (*p* = 0.0086; Figure 7C). We further analyzed the relationship between each cluster of genes within each Jλ family. Plasmablasts from HDs and HIVDs used all clusters of genes to pair with Jλ1, Jλ2, and Jλ3 in the productive repertoires (Figure 7D). There was no evidence of overutilization of any Jλ chain gene families in any repertoire (Supplementary Figures 4A–C).

### Variation in CDR3 Lengths

**Figure 8:**
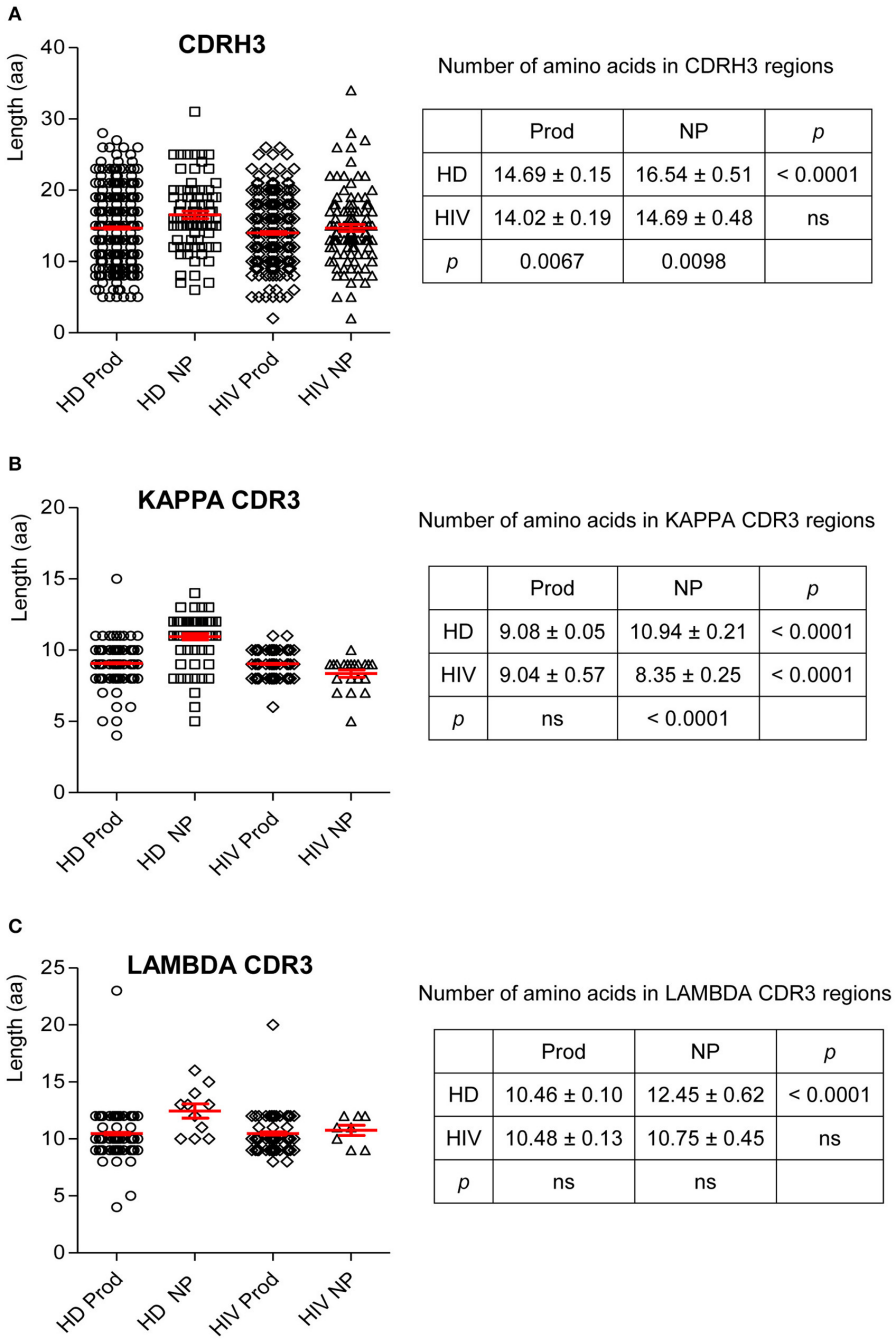
The number of amino acids in the CDR3 of H, κ, and λ chain. Unpaired two-tailed t test was used to calculate the mean and SEM for the number of amino acids in the CDR3 of H **(A)**, κ **(B)**, and λ **(C)** chains. HD, healthy donor; NP, non-productive rearrangements; Prod, productive rearrangements; HIV, HIVD; ns, not significant difference.

CDR3 length is critical in determining Ab–Ag binding characteristics. Thus, we examined the variation in CDR3 lengths on H and L chains. In HIVDs, the average length of the productive repertoire was 14.02 amino acids (aa), which was significantly shorter than that of the productive repertoire from HDs (*p* = 0.0067; Figure 8A). There was no difference in the lengths of CDRκ3 in the productive repertoires of HDs and HIVDs (Figure 8B). For HIVDs, the average CDRκ3 length was ~9 aa, which was significantly longer than that of the non-productive repertoire (*p* < 0.0001; Figure 8B). There was no notable variability in CDRλ3 length among any of the repertoires (Figure 8C).

### Amino Acid Composition of CDRH3

**Figure 9:**
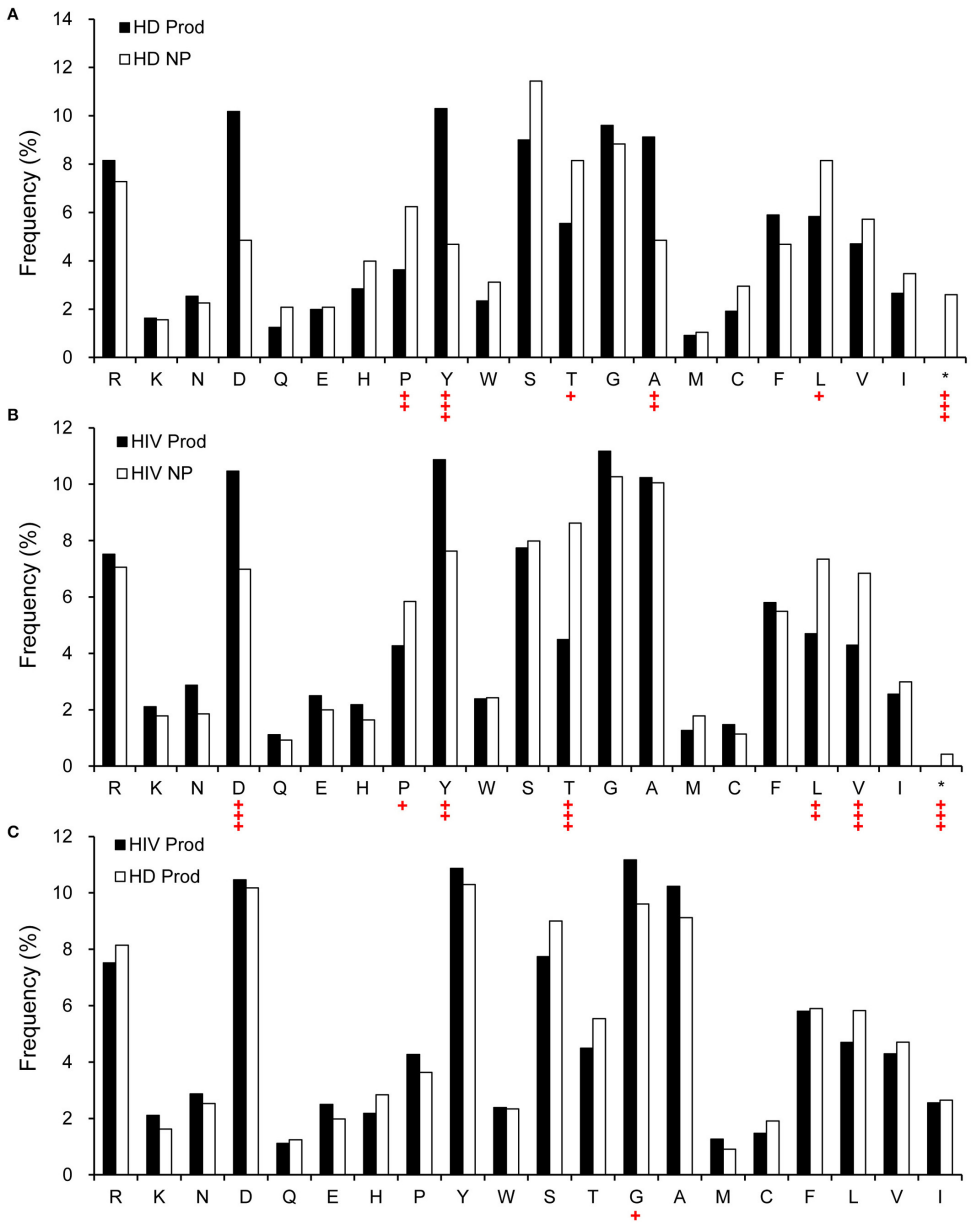
The relative amino acid usage within the CDRH3 of productive and non-productive rearrangements. **(A)** HD non-productive and productive rearrangements, **(B)** HIVD non-productive and productive rearrangements, and **(C)** HIVD productive vs. healthy donor productive CDRH3 rearrangements. *Rearrangements containing a stop codon. Chi-square or Fisher's exact tests were used, and a significant difference was considered when two-sided *p*-value < 0.05. ^+^*p* < 0.05; ^++^*p* < 0.01; ^+++^*p* < 0.0001.

Structurally, Ab CDR3 is in the center of the Ag-binding site, which interacts directly with the Ag. Therefore, we analyzed the predicted amino acid content of CDRH3 in the non-productive and productive repertoires of HIVDs and HDs. Compared to the non-productive repertoire, the productive repertoire of HIVDs showed positive selection for aspartic acid (D) (*p* < 0.0001) and tyrosine (Y) (*p* = 0.0003) and negative selection for proline (P) (*p* = 0.0188), threonine (T) (*p* < 0.0001), leucine (L) (*p* = 0.0002), and valine (V) (*p* = 0.0001) (Figure 9B). Similar trends of selection were seen in the productive repertoire of HDs when compared to the non-productive repertoire (Figure 9A). Surprisingly, there was minimal evidence of differential usage of amino acids between the productive repertoires of HIVDs and HDs, except that the former used significantly more glycine (G) (*p* = 0.0132; Figure 9C).

## Discussion

In the current study, we performed single-cell PCR and sequence analysis to examine the characteristics of plasmablast IgH and IgL in multiple HIVDs and HDs for the first time. Strikingly, our study revealed multiple differentially used genes in the comparison of the productive repertoires of HIVDs and HDs, indicating that HIV infection was the predominant factor affecting the development of the plasmablast repertoire.

During HIV infection, gp120 has long been demonstrated as a superantigen that may eliminate VH3-expressing B cells (12). Here, we observed that VH3 remained the largest family used in the productive gene rearrangements from HIVDs. A possible explanation may be that all patients involved in our study were negative in serum tests for bNAbs (7) and that non-neutralizing Abs favor the use of VH3 and share a pattern identical to Abs derived from healthy individuals (13). This observation suggests that the biased VH gene usage in the plasmablast repertoire may be related to the immune activation due to HIV infection, rather than by compensation for any depletion of VH3-expressing B cells according to the gp120 superantigen hypothesis.

We found multiple differentially used VH genes in the overall productive IGHV repertoires between HIVDs and HDs. Although limited information regarding the functionality of Abs encoded by each VH gene is available, some VH genes with differential usages have been associated with Ab specificities or disease conditions. For example, VH1-2 and VH1-46 give rise to bNAbs that recognize the CD4-binding site (14, 15), which is a promising target for elicitation by vaccination with designed immunogens. Additionally, anti-V3 mAbs exhibit a broad range of neutralizing activities (16) and preferentially use the rarely employed VH5-51 gene (17, 18). Here, the overrepresentation of VH1-2, VH1-46, and VH5-51 may reflect the potential to express bNAbs, indicating that B cells expressing such VH genes may be induced as part of an overall HIV vaccine strategy.

Interestingly, we also found several D genes that were over- or underrepresented in comparisons between the productive repertoires of HIVDs and HDs. However, the productive and non-productive repertoires of HIVDs displayed different frequencies in only one or two D genes. This is consistent with the trend observed in VH gene usages and indicates that molecular processes that take place prior to HIV infection are of limited influence in shaping the functional plasmablast repertoire during chronic HIV infection.

The recombination of D and JH genes in close proximity may occur. However, we only detected evidence of differences in the pairing of intermediate D segments (D4-11 to D2-15) and 3′ JH segments (JH1 to JH4) in non-productive rearrangements from HIVDs. Distal JH4 genes were most frequently used in all repertoires of both HDs and HIVDs. In addition, the productive repertoire of HIVDs seemed to favor the most distal JH6 genes but not the more proximal JH3. This is consistent with a previous report that repetitive rounds of D to JH recombination may occur between the common lymphoid precursor and the pre–B cell stage before a final DJH recombination (19). Unlike the H chain, the κ chain was more prone to use the proximal Jκ1 during chronic HIV infection. No evidence of overutilization of any Jλ chain gene families was detected. Taken together, our data suggest that the accessibility of V, D(H), and J gene segments during rearrangements may be determined by complex machineries and that locus proximity may not be a prominent factor affecting the molecular processes that shape the plasmablast repertoire before Ag selection in HIVDs.

A longer CDRH3 loop aids HIV bNAbs in binding to their epitopes and exhibiting neutralizing capacities (20–23). Long CDRH3 Ig sequences also exist in the HIV-negative B cell repertoire (24) and may serve as favorable targets for structure-based vaccine design. However, we found that the average CDRH3 length of the productive repertoire from HIVDs was shorter than that from HDs. It is possible that polyclonal expansion of B cells reduces overall diversity and impacts modifications in the junction region and CDRH3 length distribution.

Acidic CDRH3 regions aid anti-HIV Abs in reaching the positively charged binding cavity of gp120 (25–27). Notably, the charge, hydrophobicity, size, and shape of an Ab can be affected by the amino acid composition of CDR3s. We observed that tyrosine, glycine, alanine, and serine predominated in the productive repertoires of both study groups, consistent with the reported CDRH3 amino acid compositions of human Ig sequences (28). Furthermore, following a similar trend, both productive repertoires contained more tyrosine and fewer proline, threonine, valine, and leucine residues than the non-productive repertoires. One exception was glycine, which was positively selected in HIVDs. We speculate that this is of significance because glycine often provides high flexibility to the polypeptide chain within the CDRH3 region and thus influences Ab specificity and/or affinity. Meanwhile, increased usage of negatively charged aspartic acid was observed in the productive repertoire of HIVDs compared to the non-productive repertoire. This implies that plasmablasts encoding negatively charged CDR3 regions are positively selected in HIVDs. Overall, analysis of CDR3 lengths and amino acid compositions suggests that the choice of an individual amino acid in shaping CDR3 regions of an appropriate length and configuration is essential for the development of a functional Ig repertoire.

In the context of HIV infection, excellent studies have focused on memory B cell responses and the evolution of bNAbs (29–31). While the utility of flow cytometry–based Ig capture assay in isolating Ag-reactive plasmablasts (32) is promising, our study for the first time reveals the characteristics of the whole pool of plasmablasts in chronically HIV-infected individuals incapable of generating bnAbs through natural immunity. Due to the limited and variable numbers of sequences derived from each donor via single-cell PCR, variations between donors were seen in the current study. The different success rate of amplifying heavy and light chains led to the unequal number of sequences from single wells, which made it difficult to analyze the paired heavy and light chains instead of the whole pool of sequences. However, single-cell sorting into microwell plates is usually more feasible and less expensive, thus providing timely information (33). Therefore, our data obtained by single-cell PCR to probe the characteristics of the plasmablast repertoire will greatly facilitate future studies that may utilize more high-throughput sequencing methods to acquire more sequences to achieve a broader and deeper analysis.

The current study has several limitations. First, the HIVDs in the current study were all chronically infected with ART experience, currently off ART with the exception of one subject, and not elite controllers. Although all patients showed a comparable level of CD4 count, there were variations in the effect of ART on each patient, leading to the differences in the viral loads. In fact, some patients showed high viral load, possibly due to the inconsistency of ART. The plasmablast frequency and response may vary accordingly. Therefore, future study focused on elite controllers may provide valuable insights into the development of Ig repertoire development and bNAb against HIV. Second, considering that the number of study subjects in the current study is limited, it should be more rational to include analysis on the longitudinal samples and accomplish a binary comparison before and after HIV infection. Third, variable numbers of sequences were amplified from the study subjects; especially from some HIVDs, low numbers of sequences have been derived. This adds to another layer of variations. In addition, early studies indicate that Ig VH gene use is not random (34–39). For example, several VH genes, such as VH26, 56p1, and VH 4.21, were found to be expressed repeatedly and exceed the predicted level for random VH gene use (34–39). Gene copy number variation between individuals and across populations can play a major factor in shaping the B cell repertoire (40, 41). In the present study, interpretation of the Ig gene segment usage may be limited without determination of the copy number of the corresponding germline VH gene.

In summary, this comprehensive analysis based on a single cell clearly delineates the biased utilizations of VH and VL genes during chronic HIV infection. The results may also shed light on HIV-mediated defects in humoral responses and provide evidence for elusive bNAbs critical to the development of an effective HIV vaccine.

## Data Availability Statement

All datasets generated for this study are included in the article/Supplementary Material.

## Ethics Statement

The studies involving human participants were reviewed and approved by the University of Nebraska Medical Center. The patients/participants provided their written informed consent to participate in this study and for its publication.

## Author Contributions

HL, QZ, NJ, and ZZ designed the research. HL, SL, YYu, and YYue performed the research. KS, QZ, NJ, ZZ, and HL analyzed the data. HL, QZ, NJ, and ZZ wrote the manuscript. All authors revised the manuscript.

### Conflict of Interest

The authors declare that the research was conducted in the absence of any commercial or financial relationships that could be construed as a potential conflict of interest.

## Supplementary Material

Supplementary Figure 1 **(A)** Representative of control (top) and HIVD (bottom) showing the gating strategy used to sort single plasmablast. Digits in blue represent the percentage of plasmablast in CD19^+^ B cells. **(B)** percentages of plasmablast in CD19^+^ B cells in control donors and chronically HIV-infected individuals; Error bars indicate mean ± SEM.

Supplementary Figure 2 VH-, Vκ-, and Vλ-family distribution of RT-PCR–amplified sorted cells analyzed with IMGT/V-Quest in the productive repertoire of individual HIVD and HD. Families are color coded. The size of the colored area corresponds to the percent out of the total number of sequences, as is indicated in the center of the pie graphs. Differences in the gene family distributions were evaluated with the Chi-square test. Significant difference was considered when a two-sided *p* < 0.05.

Supplementary Figure 3 J utilization of κ locus in HD and HIVD. HD **(A)** and HIVD **(B)** non-productive (NP) and productive (Prod) rearrangements correspond to the Vκ repertoires. For all three chains, frequencies of J gene usages were also compared between the productive repertoires of HIVD and HD **(C)**. Chi-square or Fisher's exact tests were used and a significant difference was considered when a two-sided *p*-value < 0.05. ^*^*p* < 0.05; ^**^*p* < 0.01; ^***^*p* < 0.0001.

Supplementary Figure 4 J utilization of λ locus in HD and HIVD. HD **(A)** and HIVD **(B)** non-productive (NP) and productive (Prod) rearrangements correspond to the Vλ repertoires. For all three chains, frequencies of J gene usages were also compared between the productive repertoires of HIVD and HD **(C)**. Chi-square or Fisher's exact tests were used and a significant difference was considered a two-sided *p*-value < 0.05.

Supplementary Table 1 Demographics and clinical statistics of the study subjects.

Supplementary Table 2 Primers for amplification of IgH, Igκ, or Igλ genes from single plasmablast.

Supplementary Table 3 Numbers of productive and unproductive VH, Vκ, and Vλ sequences from each study subjects analyzed by IMGT/V-QUEST.

Supplementary Table 4 VDJ assignment and CDR3 amino acid sequences of polyclonal sequences derived from HIV-infected individuals determined by IMGT/V-QUEST.

Supplementary Table 5 Functional and nonfunctional sequences derived from HIVD and HD.
