# Supplementary figures and images for: Characteristics of Plasmablast Repertoire in Chronically HIV-Infected Individuals for Immunoglobulin H and L Chain Profiled by Single-Cell Analysis
Source: Front Immunol. 2020 Feb 11;10:3163. doi: 10.3389/fimmu.2019.03163 (PMC7026028; doi:10.3389/fimmu.2019.03163)

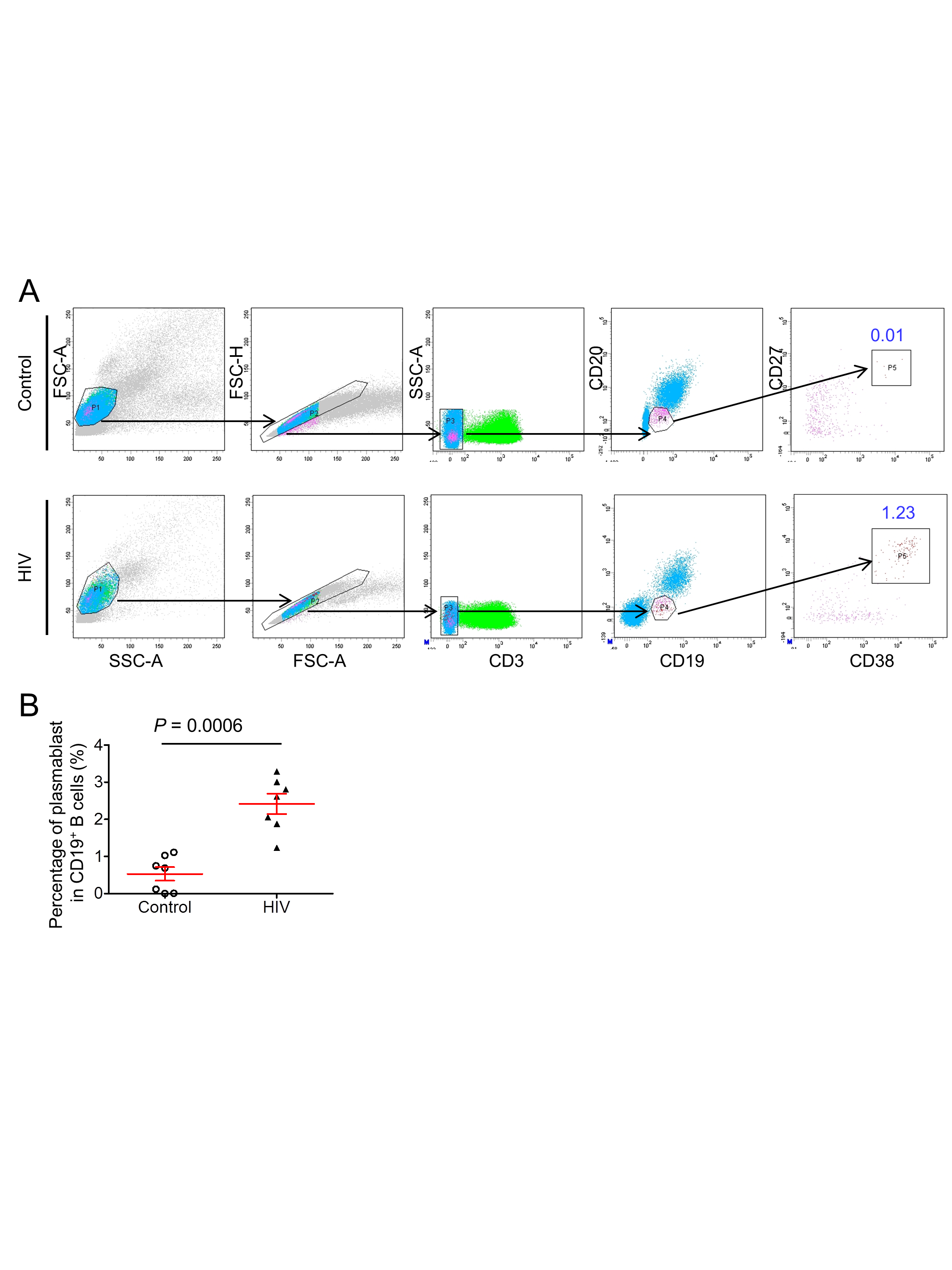

Supplement: Supplementary Figure 1 — (A) Representative of control (top) and HIVD (bottom) showing the gating strategy used to sort single plasmablast. Digits in blue represent the percentage of plasmablast in CD19+ B cells. (B) percentages of plasmablast in CD19+ B cells in control donors and chronically HIV-infected individuals; Error bars indicate mean ± SEM. [file Image_1.TIF]

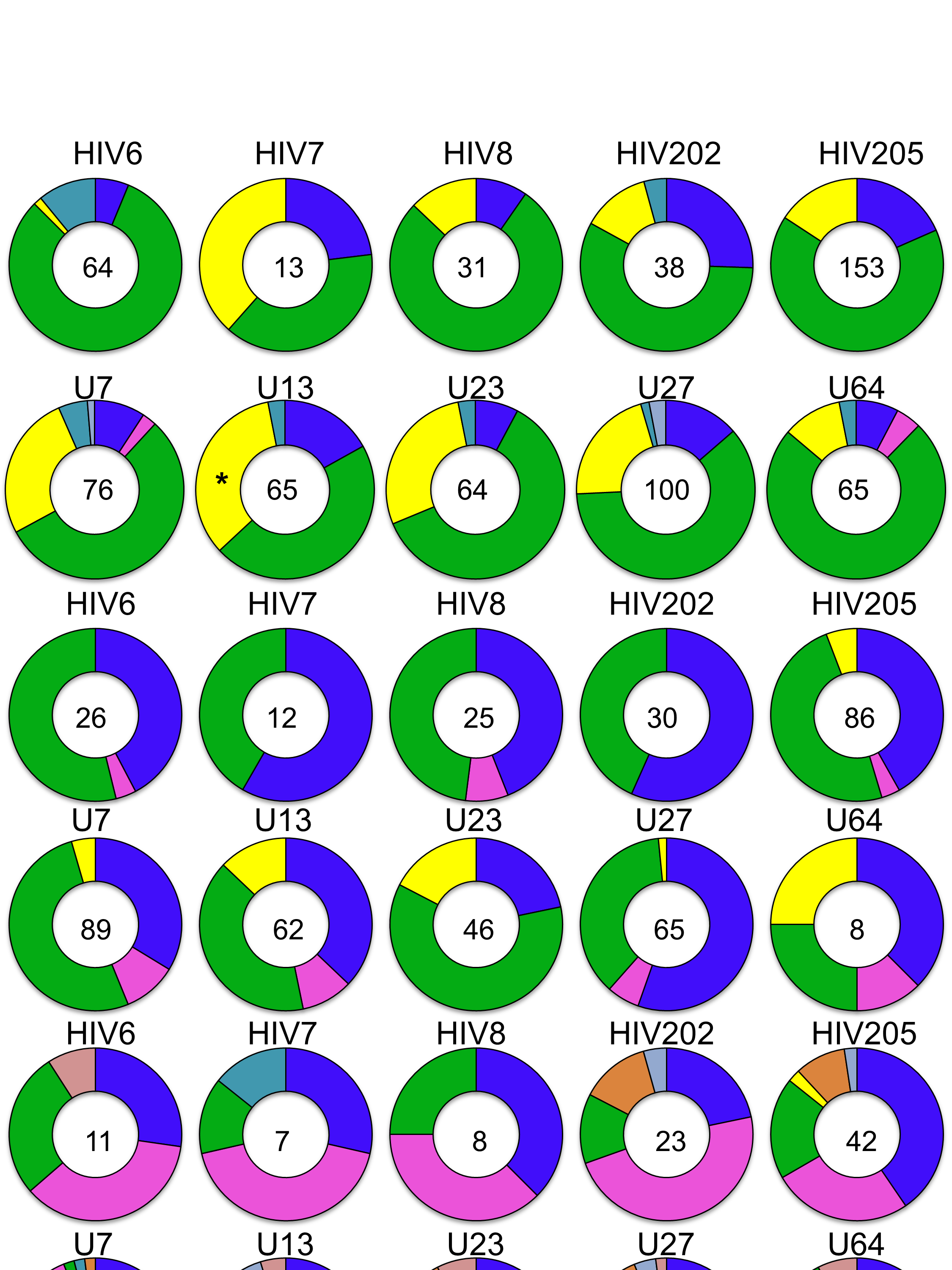

Supplement: Supplementary Figure 2 — VH-, Vκ-, and Vλ-family distribution of RT-PCR–amplified sorted cells analyzed with IMGT/V-Quest in the productive repertoire of individual HIVD and HD. Families are color coded. The size of the colored area corresponds to the percent out of the total number of sequences, as is indicated in the center of the pie graphs. Differences in the gene family distributions were evaluated with the Chi-square test. Significant difference was considered when a two-sided p < 0.05. [file Image_2.TIF]

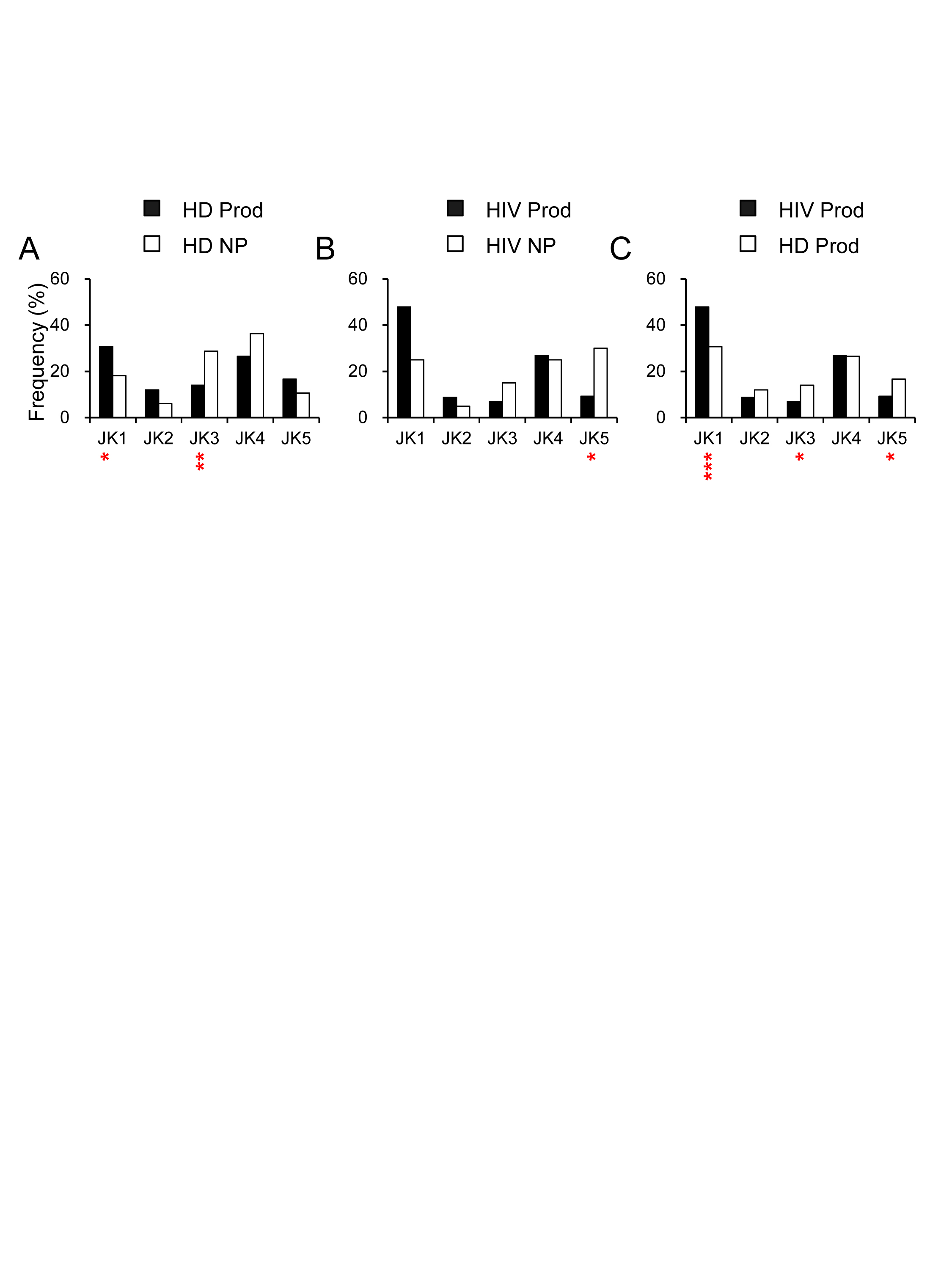

Supplement: Supplementary Figure 3 — J utilization of κ locus in HD and HIVD. HD (A) and HIVD (B) non-productive (NP) and productive (Prod) rearrangements correspond to the Vκ repertoires. For all three chains, frequencies of J gene usages were also compared between the productive repertoires of HIVD and HD (C). Chi-square or Fisher's exact tests were used and a significant difference was considered when a two-sided p-value < 0.05. *p < 0.05; **p < 0.01; ***p < 0.0001. [file Image_3.JPEG]

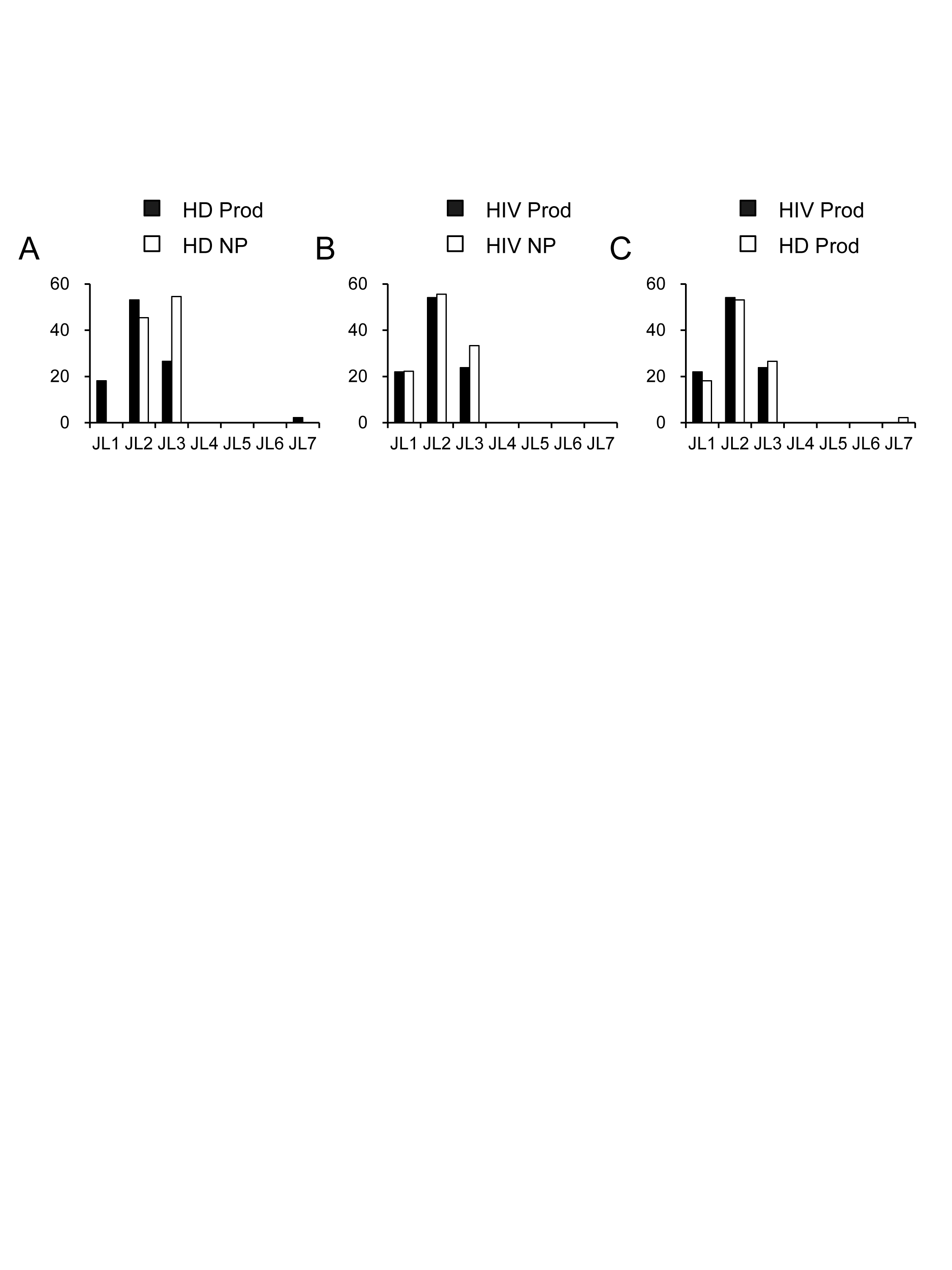

Supplement: Supplementary Figure 4 — J utilization of λ locus in HD and HIVD. HD (A) and HIVD (B) non-productive (NP) and productive (Prod) rearrangements correspond to the Vλ repertoires. For all three chains, frequencies of J gene usages were also compared between the productive repertoires of HIVD and HD (C). Chi-square or Fisher's exact tests were used and a significant difference was considered a two-sided p-value < 0.05. [file Image_4.JPEG]
